# Pharmacological rhythm control strategy and outcomes in the oldest atrial fibrillation patients: an analysis of the nationwide Italian START registry

**DOI:** 10.1093/ageing/afag157

**Published:** 2026-05-27

**Authors:** Danilo Menichelli, Gianluca Gazzaniga, Daniela Poli, Giordano Di Carlo, Emilia Antonucci, Francesco Violi, Pasquale Pignatelli, Daniele Pastori

**Affiliations:** Department of General Surgery, Surgical Specialty and Anesthesiology, University of Rome La Sapienza, Rome, Lazio, Italy; Department of General Surgery, Surgical Specialty and Anesthesiology, University of Rome La Sapienza, Rome, Lazio, Italy; Center of Atherothrombotic Disease, University Hospital Careggi, Florence, Tuscany, Italy; Postgraduate School of Internal Medicine, Department of Internal Medicine and Medical Specialties, University of Rome La Sapienza, Rome, Lazio, Italy; Arianna Anticoagulation Foundation, Bologna, Emilia-Romagna, Italy; Department of Medical and Cardiovascular Sciences, University of Rome La Sapienza, Rome, Lazio, Italy; Department of Medical and Cardiovascular Sciences, University of Rome La Sapienza, Rome, Lazio, Italy; Department of Medical and Cardiovascular Sciences, University of Rome La Sapienza, Rome, Lazio, Italy; IRCCS Istituto Neurologico Mediterraneo NEUROMED, Pozzilli, Molise, Italy

**Keywords:** atrial fibrillation, older people, antiarrhythmics, mortality

## Abstract

**Background:**

Evidence on antiarrhythmic drugs (AADs) in the oldest atrial fibrillation (AF) patients is limited. We investigated clinical characteristics and outcomes associated with AADs use in this population.

**Methods:**

The oldest (age ≥ 80 years) AF patients from the nationwide START registry were included. Patients were divided into three groups: no AADs (*n* = 3573), class 1c-AADs (*n* = 207) and Amiodarone (*n* = 464). Factors associated with AADs were evaluated using multivariable logistic regression models. The associations between AADs and all-cause mortality were assessed using Cox proportional hazards models and cardiovascular events (CVEs) were analysed using Fine-Gray competing risk models.

**Results:**

Among 4244 patients (54.9% women), the mean age was 84.8 ± 3.8 years. AADs were prescribed in 671 patients (15.8%), including amiodarone in 464 (10.9%) and 1c-AADs in 207 (4.9%). 1c-AADs use was associated with younger age and fewer comorbidities, including lower prevalence of diabetes, heart failure, chronic obstructive pulmonary disease/obstructive sleep apnoea and better functional and social status. Amiodarone use was associated with coronary artery disease and markers of frailty. Over a median follow-up of 502 (interquartile range 362–857) days, 492 deaths and 548 CVEs occurred. In unadjusted analyses, 1c-AADs were associated with lower all-cause mortality and CVEs; however, these associations were no longer significant after multivariable adjustment. Amiodarone use was not associated with clinical outcomes in either unadjusted or adjusted analyses.

**Conclusion:**

In the oldest AF patients, AADs use is influenced by comorbidity burden and frailty-related characteristics. AADs were not independently associated with mortality or CVEs, suggesting that pharmacological rhythm control may be reserved for selected cases in this population.

## Key points

- Class 1c antiarrhythmic drug (AAD) were prescribed to patients with fewer comorbidities and better functional and social status.
- Amiodarone use was associated with markers of frailty and coronary artery disease.
- In the oldest population with atrial fibrillation (AF), the use of AADs was not associated with a reduction of mortality and cardiovascular events.
- The use of AADs in the oldest AF population may be reserved in selected cases.

## Introduction

Beyond the increased thromboembolic risk that require oral anticoagulation, either with direct oral anticoagulants (DOACs) or vitamin K antagonists (VKAs), atrial fibrillation (AF) is the most common cardiac cause for urgent hospital admission in the western world, representing about 37% of cardiac hospitalizations [1]. Many of these hospital admissions are related to worsening of AF-related symptoms, rapid ventricular rate and heart failure (HF), the latter currently representing the most common cardiovascular complication in AF patients [2]. Indeed, may be associated with several symptoms as fatigue, breathlessness, palpitations and pre-syncope that could worsen the quality of life and increase the illness of these patients [3].

Symptoms burden may be obtained by an integrated management of patients with AF [4, 5] including effective rate and rhythm control strategy [3]. Evidence from a recent metanalysis showed that rhythm control strategy may lead to a reduction of cardiovascular mortality, HF events and stroke compared with a rate control strategy in randomised controlled studies [6]. However, this analysis included studies using ablation as rhythm control strategy, with most studies including patients with a mean age < 70 years [6]. Therefore, this evidence is not directly applicable to older patients aged >80 years, that have specific clinical features such as peculiar pharmacokinetics changes [7] and multiple comorbidities that could increase the proarrhythmic effect of some AADs [8, 9]. In this population, the use of AADs is therefore more controversial [10]. According to previous studies, a proportion of these patients, ranging 21%–33.5%, are on AADs [1, 11–13]. A recent metanalysis including AF patients aged 75 years or over enrolled in retrospective studies only, with high heterogeneity, and not all taking oral anticoagulants, suggested that rhythm control was associated with a lower risk of stroke, higher risk of pacemaker implantation and similar mortality compared to rate control [14]. In this analysis, different antiarrhythmic drug (AAD) drug were not analysed separately [14].

Furthermore, a rhythm control strategy may lead to a reduction of AF burden, decreasing the cognitive decline related to vascular dementia in older patients [15].

For this reason, our study aimed to: (i) identify the clinical predictors influencing the choice of AADs (1c class vs amiodarone) and (ii) evaluate the risks of all-cause mortality and cardiovascular events (CVEs) associated with the use of AADs in older AF patients.

## Methods

### START registry

The START registry is an observational, multicentre, ongoing cohort study that includes patients (aged ≥18 years) who start anticoagulation therapy, either VKAs or DOACs, throughout Italy until December 2023. Details of the START registry have been previously described [16]. The START registry has been registered on ClinicalTrials.gov (NCT02219984). The study protocol was accepted by the Institutional Review Board of each participating centre, and informed consent was obtained from patients at enrolment. The study protocol complies with the ethical guidelines of the 1975 Helsinki Declaration, and informed consent was obtained from each patient.

Only AF patients ≥80 years were included in the analysis. Patients treated with low-molecular-weight heparin were excluded as well as patients already enrolled in Phase 2 or 3 clinical studies. Patients enrolled in other observational, or Phase 4 studies were considered eligible for the study.

### Baseline characteristics

Patient’s clinical features are recorded by participants on web-based case report forms (CRF). Baseline data are demographic and clinical characteristics of patients, including type of AF [paroxysmal AF (PAF) or permanent/persistent AF (non-PAF)], cardiovascular risk factors, comorbidities such as peripheral artery disease (PAD), HF, chronic kidney disease (CKD), coronary artery disease (CAD), chronic obstructive pulmonary disease/obstructive sleep apnoea (COPD/OSAS), anaemia, diabetes, arterial hypertension, laboratory routine data, smoking habits and baseline prescribed therapy. According to European Society of Cardiology guidelines [4], we considered as AAD for the treatment of AF: Class 1c AAD such as propafenone or flecainide and Class 3 AAD (Vaughan Williams Classification) such as amiodarone. Although further drugs such as vernakalant and ibutilide were approved for the acute management of AF, their use in outpatient setting were not approved and no patients were taking them at baseline. Patients not taking any type of antiarrhythmic therapy were included in the no AAD group.

CAD was defined as history of CAD (either ischemic heart disease or coronary revascularization with stent or coronary artery bypass graft), while cerebrovascular disease is defined as previous ischemic stroke or transient ischaemic attack. CKD was defined according to an estimated glomerular filtration rate <60 ml/min. PAD was defined according to European Society of Cardiology (ESC) guidelines [17].

### Study endpoints

The endpoints of the study were (i) to investigate factors associated with the choice of AAD and (ii) to evaluate the association between AAD use with adverse clinical events such as all-cause mortality and CVEs in the oldest cohort.

### Statistical analysis

Continuous variables were reported as mean and standard deviation and group comparisons were performed by unpaired Student’s *t-*test or ANOVA if the variable has a normal distribution at Kolmogorov–Smirnov test. Conversely, continuous variables were reported as median and interquartile ranges (IQR) and group comparison were performed by Mann–Whitney test. Proportions and categorical variables were tested by the chi-squared test.

Population characteristics have been described according to the use of AADs. A multivariable stepwise logistic regression to establish clinical factors associated with Class 1c and amiodarone use was performed including all variables evaluated at baseline. Results of the multivariable logistic regression were expressed as odds ratio and 95% confidence interval (95% CI).

Univariable and multivariable Cox proportional hazards regression analysis was used to calculate the adjusted relative hazard ratios (HRs) and 95% CI of all-cause of death by each clinical variable. In the multivariable analysis, individual variables were entered in the model.

The Fine-Gray model was used to estimate the cumulative incidence of CVEs while accounting for the competing risk of all-cause mortality. A univariate model was first fitted, accounting for AAD (reference: no AAD). Subsequently, an adjusted multivariate model was performed, incorporating baseline demographic and clinical covariates. Results were expressed as subdistribution HR (sHR) and 95% CI.

A subgroups analysis was performed according to AF phenotype (PAF and non-PAF), concomitant use of beta-blockers and concomitant CAD. For all-cause mortality endpoint, univariable Cox proportional hazards regression analysis was performed for each clinical variable, then a multivariable Cox proportional hazards regression analysis model was built adding each variable with a significant association with clinical endpoints. For CVEs endpoint, univariable Fine-Gray analysis was performed for each clinical variable, then a multivariable Fine-Gray analysis model was built adding each variable with a significant association with clinical endpoints.

Only *P* values <.05 were considered as statistically significant. All tests were two-tailed, and analyses were performed using computer software packages (IBM SPSS-25, SPSS Inc., R Software v. 4.2.3 and MedCalc).

## Results

### Population characteristics

**Figure 1:**
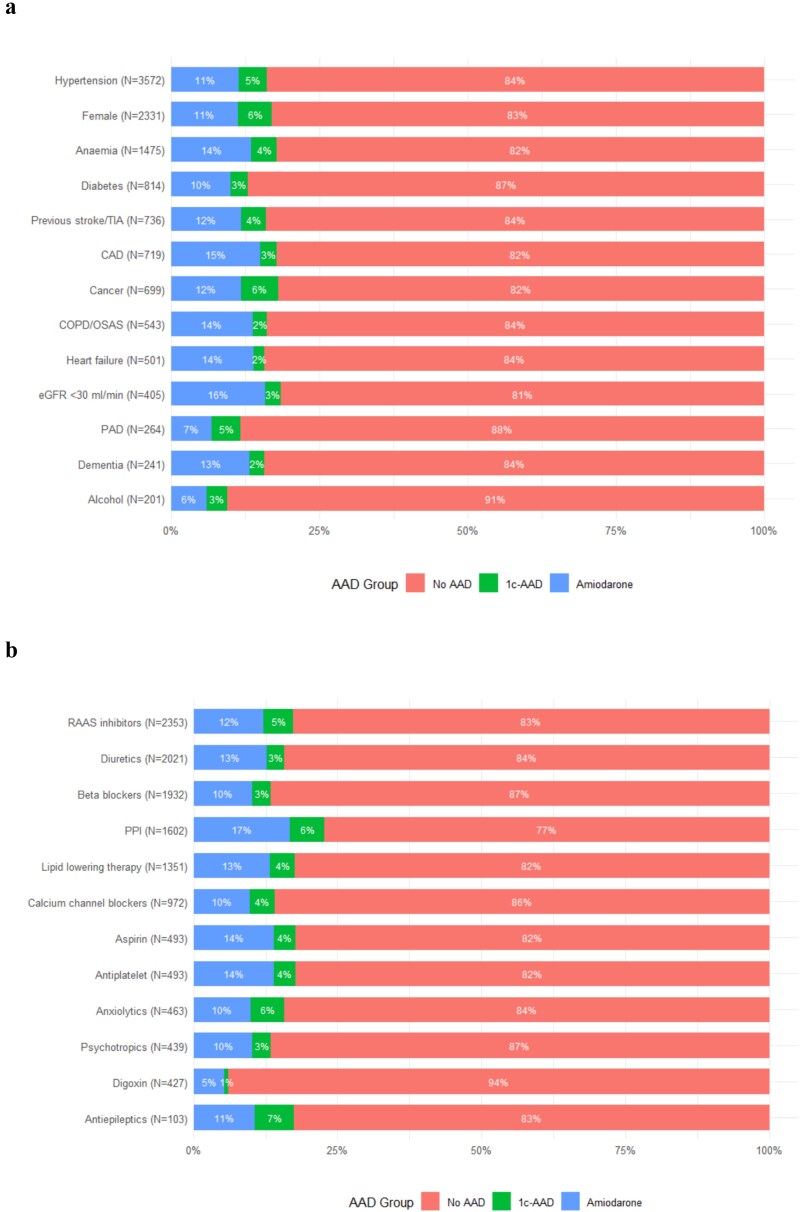
Use of AADs stratified according to comorbidities (Panel A) and concomitant treatment (Panel B). eGFR, estimated glomerular filtration rate; PPI, proton pump inhibitors.

We included in the analysis 4244 AF patients, of whom 671 (15.8%) were prescribed with AADs. Of them, 207 (4.9%) were taking 1c-AAD and 464 (10.9%) were taking amiodarone. No patients were taking both 1c-AAD and amiodarone. Mean age was 84.8 ± 3.8 years, 54.9% were women and 30.9% had PAF (Table 1). Overall, patients taking 1c-AAD and amiodarone were with a higher prevalence of PAF compared to patients without AAD. Patients taking 1c-AAD were more frequently women and had a lower proportion of patients with history of CAD, diabetes mellitus, HF, COPD/OSAS and treated with digoxin or betablockers compared to no AAD cohort (Table 1). On the other hand, patients taking amiodarone were more frequently affected by comorbidities such as history of CAD, COPD/OSAS and anaemia compared to no AAD cohort. A detailed description of population characteristics according to AAD administered was reported in Table 1. Proportion of patients taking each AADs seems to be influenced by concomitant comorbidities and therapy as shown in Figure 1, Panel A and B, respectively.

**Table 1:** Patients’ characteristics according to AADs.

	Total cohort (*n*: 4244)	No AAD (*n*: 3573)	1c-AAD (*n*: 207)	Amiodarone (*n*: 464)	Overall *P* value	*P*-value *No AAD vs 1c-AAD*	*P*-value *No AAD vs Amiodarone*
**Age (years)**	84.8 ± 3.8	84.9 ± 3.8	83.5 ± 3.2	84.3 ± 3.5	<.001	<.001	.037
**Women (%)**	2331 (54.9)	1934 (54.1)	133 (64.3)	264 (56.9)	.012	.004	.260
**Paroxysmal AF (%)**	1300 (30.9)	967 (27.3)	128 (62.7)	205 (44.5)	<.001	<.001	<.001
**Arterial hypertension (%)**	3572 (84.2)	2997 (83.9)	165 (79.7)	410 (88.4)	.009	.115	.012
**Diabetes (%)**	814 (19.2)	709 (19.8)	23 (11.1)	82 (17.7)	.006	.002	.269
**Previous CAD (%)**	720 (17.0)	591 (16.5)	20 (9.7)	108 (23.3)	<.001	.009	<.001
**Previous cerebrovascular disease (%)**	736 (17.3)	618 (17.3)	31 (15)	87 (18.8)	.483	.389	.438
**Obesity** ^**a**^ **(%)**	623 (14.7)	533 (14.9)	25 (12.1)	65 (14.0)	.485	.263	.604
**Anaemia (%)**	1475 (34.8)	1213 (33.9)	62 (30)	200 (43.1)	<.001	.237	<.001
**HF (%)**	1101 (25.9)	943 (26.4)	19 (9.2)	139 (30.0)	<.001	<.001	.103
**Cancer (%)**	699 (16.5)	573 (16.0)	43 (20.8)	83 (17.9)	.139	.073	.309
**Peripheral artery disease (%)**	264 (6.2)	233 (6.5)	13 (6.3)	18 (3.9)	.086	.891	.027
**COPD/OSAS (%)**	543 (12.8)	455 (12.7)	13 (6.3)	75 (16.2)	.002	.006	.040
**CKD** ^**b**^ **(%)**	1000 (23.6)	881 (24.7)	58 (28.2)	61 (13.1)	<.001	.263	<.001
**Alcohol use (%)**	201 (4.7)	182 (5.1)	7 (3.4)	12 (2.6)	.037	.272	.018
**CHA**_**2**_ **DS**_**2**_ **-VASc (mean)**	4.3 ± 1.2	4.3 ± 1.2	4.0 ± 1.2	4.5 ± 1.3	<.001	.011	.425
**HAS-BLED (mean)**	2.3 ± 0.7	2.3 ± 0.7	2.1 ± 0.7	2.4 ± 0.8	<.001	.019	.037
***Frailty elements***							
**Pre-existing dementia (%)**	241 (5.7)	203 (5.7)	6 (2.9)	32 (6.9)	.118	.088	.294
**Immobilisation syndrome (%)**	60 (1.4)	50 (1.4)	2 (1.0)	8 (1.7)	.732	.603	.580
**Wheelchair users (%)**	207 (4.9)	169 (4.7)	3 (1.4)	35 (7.5)	.002	.028	.009
**Tendency to fall (%)**	148 (3.5)	127 (3.6)	6 (2.9)	15 (3.2)	.839	.619	.723
**Living alone (%)**	260 (6.1)	228 (6.4)	17 (8.2)	15 (3.2)	.013	.298	.007
**Social/familial support (%)**	3059 (72.1)	2498 (69.9)	169 (81.6)	392 (84.5)	<.001	<.001	<.001
***Therapy***							
**Use of DOAC (%)**	2716 (64.0)	2276 (63.7)	154 (74.4)	286 (61.6)	.004	.002	.386
**Type of DOAC**							
*Apixaban*	950 (22.4)	918 (35.9)	48 (31.2)	84 (29.5)	.270	.436	.145
*Rivaroxaban*	645 (15.2)	540 (23.7)	34 (22.1)	71 (24.9)			
*Edoxaban*	615 (14.5)	499 (21.9)	41 (26.6)	75 (26.3)			
*Dabigatran*	506 (11.9)	420 (18.5)	31 (20.1)	55 (19.3)			
**Concomitant Aspirin (%)**	493 (11.6)	406 (11.4)	18 (8.7)	69 (14.9)	.035	.237	.027
**Lipid lowering therapy (%)**	1351 (31.8)	1114 (31.2)	57 (27.5)	180 (38.8)	.002	.271	.001
**RAAS inhibitors (%)**	2353 (55.4)	1946 (54.5)	120 (58.0)	287 (61.9)	.008	.324	.003
**Beta blockers (%)**	1932 (45.5)	1673 (46.8)	62 (30.0)	197 (42.5)	<.001	<.001	.076
**Calcium channel blockers (%)**	972 (22.9)	835 (23.4)	42 (20.3)	95 (20.5)	.248	.307	.163
**Diuretics (%)**	2021 (47.6)	1703 (47.7)	61 (29.5)	257 (55.4)	<.001	<.001	.002
**Digoxin (%)**	427 (10.1)	401 (11.2)	3 (1.4)	23 (5.0)	<.001	<.001	<.001
**Propafenone (%)**	63 (1.5)	0 (0.0)	63 (30.4)	0 (0.0)	–	–	–
**Flecainide (%)**	144 (3.4)	0 (0.0)	144 (69.6)	0 (0.0)	–	–	–
**Proton pump inhibitors (%)**	1602 (37.7)	1238 (43.6)	95 (45.9)	269 (58.0)	<.001	.001	<.001
**Antipsychotic drugs (%)**	439 (10.3)	380 (10.6)	14 (6.8)	45 (9.7)	.183	.076	.536
**Anxiolytic drugs (%)**	463 (10.9)	390 (10.9)	27 (13.0)	46 (9.9)	.486	.342	.513
**Antiepileptic drugs (%)**	103 (2.4)	85 (2.4)	7 (3.4)	11 (2.4)	.658	.363	.991

^a^Defined as body mass index ≥ 30 kg/m^2^.

^b^Defined as estimated glomerular filtration rate (eGFR) < 60 ml/min.

### Predictors of AAD administration

We found an inverse association between age, female sex, the coexistence of cardiovascular risk factors and comorbidities as diabetes mellitus, HF, COPD/OSAS and the use of Class 1c AAD (Table 2, Panel A). Conversely, the presence of PAF was associated with the use of Class 1c AAD. Of note, the use of beta-blockers, digoxin, an add-on rate control drug, the use of antipsychotic drugs, lipid lowering therapy and diuretics were inversely associated with Class 1c AAD, while the use of DOACs (over VKAs) and proton pump inhibitors was preferred in patients taking these drugs (Table 2, Panel A).

**Table 2:** Multivariable stepwise logistic regression of factors associated with use of Class 1c (Panel A) and Amiodarone (Panel B) AADs.

	OR	95% CI		*P*-value
		Low	High	
**A—Class 1c AAD**				
**Age**	0.90	0.86	0.94	<.001
**Female sex**	0.64	0.47	0.87	.004
**Paroxysmal AF**	3.23	2.40	4.35	<.001
**Diabetes mellitus**	0.60	0.38	0.95	.031
**HF**	0.45	0.28	0.75	.002
**COPD/OSAS**	0.52	0.29	0.94	.031
**No Family/social support**	0.52	0.36	0.76	<.001
**DOAC *vs. VKA***	1.45	1.04	2.03	.030
**Lipid lowering therapy**	0.71	0.51	0.99	.045
**Diuretics**	0.61	0.44	0.85	.003
**Digoxin**	0.21	0.07	0.66	.008
**PPI**	1.73	1.28	2.36	<.001
**Beta blockers**	0.53	0.38	0.73	<.001
**Antipsychotic drugs**	0.50	0.28	0.91	.023
**B—Amiodarone**				
**Age**	0.94	0.91	0.96	<.001
**Paroxysmal AF**	1.91	1.56	2.35	<.001
**CAD**	1.37	1.07	1.75	.013
**PAD**	0.48	0.29	0.80	.005
**CKD**^**a**^	0.41	0.31	0.55	<.001
**Wheelchair**	1.53	1.02	2.28	.038
**Living alone**	0.55	0.32	0.94	.030
**No Family/social support**	0.55	0.42	0.72	<.001
**Diuretics**	1.32	1.07	1.62	.009
**Digoxin**	0.42	0.27	0.65	<.001
**PPI**	2.17	1.76	2.68	<.001
**Beta-blockers**	0.75	0.61	0.93	.007

eGFR, estimated glomerular filtration rate; PPI, proton pump inhibitors.

^a^Defined as eGFR < 60 ml/min.

On the other hand**,** age, CKD and PAD were inversely associated with amiodarone administration (Table 2, Panel B), while history of CAD and paroxysmal AF were directly associated with it. Digoxin and beta blockers were also inversely associated with amiodarone use, while diuretics and proton pump inhibitors were directly associated with amiodarone use.

Of note, the presence of clinical frailty elements such as the use of wheelchair was associated with the use of amiodarone, while clinical sign of residual autonomy as the possibility of living alone and the absence of family support were inversely associated (Table 2, Panel B).

### A‌AD administration, all-cause mortality and CVEs risk

Over a median follow-up of 502 (IQR 362–857) days, 492 all-cause deaths and 548 CVEs occurred. At univariable Cox regression analysis, the use of Class 1c AAD, but not the use of amiodarone, was associated with a lower risk of all-cause death (Table 3, Panel A). Similarly, univariable Fine-Gray analysis showed an association only between Class 1c AAD and CVEs accounting for the competing risk of all-cause mortality (Table 3, Panel B). These associations observed may be explained by a potential selection effect due to a higher prescription of 1c-AADs than amiodarone in healthier patients.

**Table 3:** Association between AAD use and outcomes: univariable cox regression for all-cause mortality (Panel A); univariable Fine-Gray competing risks analysis for cardiovascular events (Panel B).

**A—All-cause mortality**	**HR**	**95% CI**		***P*-value**
**Class 1c *vs No antiarrhythmics***	0.37	0.19	0.72	.003
**Amiodarone *vs No antiarrhythmics***	1.10	0.83	1.45	.513
**B—CVEs**	**sHR**	**95% CI**		***P*-value**
**Class 1c *vs No antiarrhythmics***	0.44	0.25	0.79	.005
**Amiodarone *vs No antiarrhythmics***	1.04	0.79	1.36	.797

However, at multivariable analysis adjusted for comorbidities and potential confounders, Class 1c AAD use was not associated with all-cause of death (HR 0.64, 95% CI 0.33–1.26, *P* = .200) and CVEs (sHR 0.70. 95% CI 0.39–1.27, *P* = .239). Similar results were found for amiodarone (Table 4, Panel A and B).

**Table 4:** Multivariable cox regression analysis of factors associated with all-cause death (Panel A) and Fine and Gray competing risks analysis of cardiovascular events (Panel B).

Panel A	HR	95% CI		*P*-value
		Low	High	
**Age (years)**	1.12	1.09	1.15	<.001
**Female sex**	0.70	0.57	0.85	<.001
**Paroxysmal AF**	0.82	0.66	1.02	.074
**Arterial hypertension**	0.96	0.73	1.27	.771
**Diabetes**	1.21	0.96	1.52	.107
**Coronary artery disease**	1.39	1.09	1.77	.007
**Peripheral artery disease**	1.55	1.12	2.13	.008
**Obesity**	1.14	0.86	1.49	.363
**Anaemia**	1.29	1.06	1.56	.009
**HF**	1.15	0.93	1.42	.187
**Cancer**	1.05	0.83	1.33	.706
**Previous cerebrovascular disease**	1.24	0.99	1.56	.067
**COPD/OSAS**	1.68	1.33	2.12	<.001
**CKD** ^**a**^	0.69	0.52	0.91	.008
**Alcohol**	0.89	0.57	1.39	.611
**Dementia**	1.40	0.99	1.98	.054
**Wheelchair users**	1.67	1.18	2.37	.004
**Immobilisation syndrome**	0.99	0.48	2.06	.979
**Tendency to fall**	0.86	0.54	1.36	.507
**Living alone**	0.85	0.57	1.29	.448
**Social/familial support**	0.81	0.65	1.02	.067
**Class 1c *vs* No AAD**	0.64	0.33	1.26	.200
**Amiodarone *vs* No AAD**	1.12	0.84	1.50	.453
**DOAC**	0.35	0.28	0.43	<.001
**Concomitant Aspirin**	0.83	0.62	1.11	.198
**Lipid lowering therapy**	0.68	0.54	0.85	.001
**RAAS inhibitors**	0.80	0.66	0.97	.020
**Beta blockers**	0.99	0.82	1.20	.931
**Calcium channel blockers**	1.08	0.86	1.34	.513
**Diuretics**	1.02	0.83	1.25	.889
**Digoxin**	1.16	0.88	1.53	.281
**Proton pump inhibitors**	1.10	0.90	1.35	.334
**Antipsychotic drugs**	1.31	0.96	1.77	.088
**Anxiolytic drugs**	1.03	0.78	1.37	.824
**Antiepileptic drugs**	0.90	0.48	1.71	.755
Panel B	sHR	95% CI		*P*-value
		**Low**	**High**	
**Age (years)**	1.10	1.07	1.13	<.001
**Female sex**	0.69	0.57	0.83	<.001
**Paroxysmal AF**	0.86	0.70	1.06	.161
**Arterial hypertension**	0.93	0.72	1.21	.605
**Diabetes**	1.21	0.97	1.50	.090
**Coronary artery disease**	1.35	1.07	1.70	.011
**Peripheral artery disease**	1.64	1.22	2.21	.001
**Obesity**	1.09	0.84	1.42	.511
**Anaemia**	1.26	1.05	1.51	.012
**HF**	1.19	0.97	1.45	.094
**Cancer**	1.08	0.86	1.34	.525
**Previous cerebrovascular disease**	1.30	1.05	1.61	.017
**COPD/OSAS**	1.66	1.33	2.07	<.001
**CKD** ^**a**^	0.62	0.48	0.81	<.001
**Alcohol**	1.15	0.79	1.68	.468
**Dementia**	1.34	0.96	1.86	.086
**Wheelchair users**	1.58	1.13	2.20	.008
**Immobilisation syndrome**	0.96	0.48	1.92	.907
**Tendency to fall**	0.94	0.61	1.43	.757
**Living alone**	0.85	0.58	1.26	.420
Panel B	sHR	95% CI		*P*-value
		Low	High	
**Social/familial support**	0.78	0.64	0.96	.020
**Class 1c *vs* No AAD**	0.70	0.39	1.27	.239
**Amiodarone *vs* No AAD**	1.03	0.77	1.36	.856
**DOAC**	0.46	0.38	0.56	<.001
**Concomitant Aspirin**	0.94	0.72	1.22	.631
**Lipid lowering therapy**	0.71	0.58	0.88	.002
**RAAS inhibitors**	0.79	0.66	0.95	.013
**Beta blockers**	0.94	0.78	1.12	.463
**Calcium channel blockers**	1.03	0.83	1.27	.791
**Diuretics**	0.98	0.81	1.19	.835
**Digoxin**	1.15	0.88	1.49	.311
**Proton pump inhibitors**	1.05	0.87	1.27	.599
**Antipsychotic drugs**	1.27	0.95	1.69	.110
**Anxiolytic drugs**	1.05	0.80	1.38	.727
**Antiepileptic drugs**	0.88	0.48	1.61	.675

^a^Defined as estimated glomerular filtration rate (eGFR) < 60 ml/min.

Age and comorbidities as CAD, PAD, anaemia and COPD/OSAS were strongly associated with both all-cause mortality and CVEs (Table 4, Panel A and B). In addition, a previous history of cerebrovascular disease was associated with a higher risk of CVEs. Conversely, female sex and the use of DOACs (vs VKAs), the use of lipid lowering therapy and renin-angiotensin-aldosterone (RAAS) inhibitors were associated with a lower risk of both all-cause mortality and CVEs. Finally, a clinical frailty element as the use of wheelchair was associated with a higher risk of all-cause of death and CVEs (Table 4, Panel A and B).

### Subgroup analysis

#### PAF vs non-PAF patients

During follow-up, 381 deaths and 417 CVEs occurred in the non-PAF group, while 111 all-cause of death and 131 CVEs occurred in the PAF group. Univariable and multivariable Cox analysis for all-cause mortality according to PAF/non-PAF group is shown in Supplementary Table SS1. At multivariable Cox analysis, both Class 1c AADs and amiodarone were not associated with all-cause mortality. In the PAF subgroup, older age, PAD, anaemia, COPD/OSAS, dementia, the use of digoxin, anxiolytic drugs and proton pump inhibitors were associated with a higher risk of all-cause mortality, while the use of DOACs was associated with a lower risk. Conversely, in the non-PAF subgroup, older age and comorbidities as CAD, PAD, anaemia, COPD/OSAS were associated with higher risk of all-cause mortality, while female sex, DOAC, lipid lowering therapy and RAAS inhibitors use were associated with a lower risk.

Subsequently, univariable and multivariable Fine-Gray analysis for CVEs accounting for the competing risk of all-cause mortality according to PAF/non-PAF group was shown in the Supplementary Table SS2. At multivariable Fine-Gray analysis, both Class 1c AADs and amiodarone were not associated with CVEs. In the PAF subgroup, older age, PAD, COPD/OSAS, digoxin and antipsychotic drugs were associated with higher risk of CVEs, while DOAC use was associated with a lower risk. On the other hand, in the non-PAF group, older age, comorbidities as CAD, anaemia, COPD/OSAS and clinical frailty elements as wheelchair use were associated with higher risk of CVEs, while the use of DOAC, lipid lowering therapy and RAAS inhibitors and female sex were associated with a lower risk of CVEs.

#### Beta-blockers use

During follow-up, among 1932 patients receiving beta-blockers, 252 deaths and 272 CVEs occurred, while among 2312 patients not receiving beta-blockers, 240 deaths and 276 CVEs were observed. Results of univariable and multivariable Cox regression analyses for all-cause mortality stratified by beta-blocker use are presented in Supplementary Table SS3. In both subgroups, Class 1c and amiodarone were not associated with all-cause mortality after multivariable adjustment. Factors independently associated with mortality in both groups included age, COPD/OSAS, and use of VKA instead of DOAC.

Fine-Gray analyses for CVEs in beta-blocker users and non-users (Supplementary Table SS4) showed consistent findings: age, COPD/OSAS and VKA use were significantly associated with higher risk, while AAD class was not.

#### Concomitant CAD

During follow-up, among 719 patients with CAD, 127 deaths and 116 CVEs occurred, compared with 376 deaths and 421 CVEs among 3525 patients without CAD. Cox regression analyses for all-cause mortality in patients with and without CAD are presented in Supplementary Table SS5. In both subgroups, AAD class was not significantly associated with mortality after adjustment. Similarly, Fine-Gray competing risk analyses for CVEs (Supplementary Table SS6) showed no association between AAD class and increased CVE risk, irrespective of CAD status. Across both analyses, age and VKA use (vs. DOAC) consistently emerged as predictors of adverse outcomes.

## Discussion

Our study performed on a real-world population of the oldest AF patients taking oral anticoagulants enrolled from multiple centres, we found that AADs were used in a significant proportion of patients aged 80 years or more. The pattern of AADs prescription was influenced by comorbidities, frailty elements and concomitant therapy; Class 1c AADs were preferred in patients with less comorbidities, better performance and social status. We did not find any association between AAD use and CVEs and all-cause mortality reduction at multivariable analysis. However, potential non-cardiovascular benefits as reduction in decline cognition and improvement in quality of life, not explored in this study, may justify the selective use of AADs even in the absence of a reduction in mortality and CVEs in selected older patients with AF [18].

The prevalence of older patients treated with AADs in our cohort was 15.8%; this proportion is highly variable among studies, probably depending on different clinical characteristics of patients [1, 11–13].

In a cohort of 20 172 AF Chinese patients [11] the overall prevalence of AADs use was 36.3%, with this proportion decreasing to 21% in the older group aged ≥75 years. A higher proportion of patients taking AADs was also observed in the Outcomes Registry for Better Informed Treatment of Atrial Fibrillation [12]: in this cohort including 10 132 patients with AF, 29% of patients were taking AADs at baseline, however, also in this trial the median age (75 years) was lower compared to our cohort and a higher proportion of PAF patients were enrolled (51%). Unfortunately, the lack of data in these studies about type of AADs, the drugs used for rate control strategy did not allow further comparison.

We first investigated clinical factors associated with the prescription of different AADs in this cohort. We found that patients advancing age and paroxysmal AF were associated with AADs use independently from the type of drug.

Patients prescribed on Class 1c AADs, were less likely to be women, with diabetes, HF and COPD/OSAS. The inverse association with female sex is concerning considering that women are more likely to experience AF-related symptoms than men [19] and previous evidence suggest that women are usually treated more conservatively especially with less cardioversion or ablation [20]. This gender-gap in the treatment of female AF patients need attention.

We also found an inverse association between class 1c prescription and the use of antipsychotic drugs suggesting a cautious use of this potentially harmful drug combination by physicians [21, 22].

Finally, an inverse association between HF and use of Class 1c AADs, and a direct association between amiodarone and CAD were found; this could be explained by the contraindication to use Class 1c in patients with structural or ischemic heart disease, preferring amiodarone.

Regarding social determinants/frailty elements, that were very common in the eldest AF patients [23], we found that rhythm control strategy was generally preferred in patients with social or family support, but the use of amiodarone was more prevalent in patients using wheelchairs probably reflecting a more fragile patient, as also shown by CAD prevalence. These findings suggest that in addition to clinical characteristics, family and social factors may influence strategies treatment.

Regarding clinical outcomes, we found no association between AADs and mortality and CVEs after adjustment for clinical factors. There are not many studies investigating clinical outcomes in older patients according to AAD use. The previously mentioned metanalysis including nearly 100 000 AF patients from 7 retrospective studies found a similar all-cause and cardiovascular mortality for rhythm vs rate control strategy [14]. However, these results should be interpreted with caution for several reasons. As mentioned, retrospective design of the included studies is an intrinsic bias of the analysis. Moreover, many of these studies did not include patients on DOACs or included patients not treated with oral anticoagulants, all factors affecting mortality rate in the AF population. The study by Ionescu-Ittu *et al.*, despite including patients in the pre-DOAC era and nearly 40% not taking any anticoagulants, showed that the benefit of rhythm control strategy became evident after 4 years of follow-up [24]. This lag in the beneficial effect of rhythm control strategy may not be beneficial in the oldest patients or in those with limited life expectancy.

Conversely, in the sub-analysis of Atrial Fibrillation Follow-up Investigation of Rhythm Management randomised trial, according to age ≥65 years, rhythm control strategy was associated with a worse prognosis and higher risk of death compared to rate control one [25, 26]. This is probably because all patients were on warfarin and while continuous anticoagulation was mandatory in the rate control group, it could be stopped at physician’s discretion in the rhythm control group if sinus rhythm was restored after at least 4 weeks [25, 26]. This may have caused an excess of mortality in that treatment arm.

### Strengths

Our study has several strengths. Firstly, we evaluated an older AF population from a multicentre real-world study highlighting the real use of AADs in this clinical setting. We also analysed separately Class 1 c and amiodarone describing patterns of prescription in this high-risk subgroup of patients. Our study showed no clinical benefits of AADs as strategy in reducing CVEs and all-cause mortality. This is a fundamental issue in older patients, in whom the prescribing of not useful drugs that potentially could increase adverse effects and harmful drug–drug interactions. We stratified survival analysis for the use of beta blockers or the presence of CAD, which are both highly prevalent in older patients finding similar results. Finally, a competing risks analysis approach was used to evaluate CVEs.

### Limitations

Several limitations should be acknowledged. First, the study cohort predominantly consisted of Caucasian patients due to the demographic characteristics of the participating centres, limiting the generalizability of our findings to other ethnic groups. In addition, as an observational study, we cannot exclude the presence of unintended biases or infer causality in the associations observed. Besides, in the START registry several variables such as heart rate during follow-up, AF burden, quality of life estimated by validated score as European Heart Rhythm Association (EHRA) score, and cognitive outcomes were not evaluated. These variables may represent ‘soft’ benefits during a rhythm control strategy with AADs and may be a potential indication for AADs use in selected older AF patients. Furthermore, no data about other strategies of rhythm control such as cardioversion and catheter ablation were available in our cohort. This may be cause by the low prevalence of these procedures in the older population and a lower enrolment that make this population underrepresented and these procedures undervalued in common clinical practice.

In addition, a limitation of our study was the lack of data on drug doses or drug titration/discontinuation during follow-up. Finally, we have no data on adverse effects of AADs administered, reducing the global evaluation of clinical benefit related to these drugs.

Despite these limitations, our findings contribute to the growing body of evidence supporting a more cautious use of AADs in older patients with AF, emphasising the lack of clinical benefit on mortality and CVEs and the need for a tailored therapy based on patient-specific factors.

## Conclusions

Among older AF patients, Class 1c AADs were prescribed in patients with fewer comorbidities and better functional and social status. These results indicate that frailty elements affect the choice of AADs in older AF patients. Furthermore, Class 1c agents were associated with favourable outcomes in unadjusted models, but, as for amiodarone, these effects did not persist after adjustment for comorbidities. Due to potential adverse effects the use of AADs may be limited to selected cases of patients with uncontrolled symptoms.

## Supplementary Material

aa-26-0512-File002_afag157

## Data Availability

Data supporting the findings of this study are available from the corresponding author upon reasonable request.
