# Supplementary material for: Pharmacological rhythm control strategy and outcomes in the oldest atrial fibrillation patients: an analysis of the nationwide Italian START registry
Source: Age Ageing. 2026 May 27;55(5):afag157. doi: 10.1093/ageing/afag157 (PMC13215591; doi:10.1093/ageing/afag157)
Supplement: aa-26-0512-File002_afag157 [file aa-26-0512-file002_afag157.docx]

**Pharmacological rhythm control strategy and outcomes in the oldest atrial fibrillation patients: an analysis of the nationwide Italian START registry.**

*Supplementary material*

## Supplementary Table 1. Univariable and Multivariable Cox regression analysis of factors associated with all-cause mortality in patients with paroxysmal atrial fibrillation (PAF) and persistent/permanent atrial fibrillation (non-PAF).

## Supplementary Table 2. Univariable and Multivariable Fine-Gray analysis of factors associated with cardiovascular events (CVEs) in patients with paroxysmal atrial fibrillation (PAF) and persistent/permanent atrial fibrillation (non-PAF).

## Supplementary Table 3. Univariable and Multivariable Cox regression analysis of factors associated with all-cause mortality in patients with and without beta-blocker therapy.

## Supplementary Table 4. Univariable and Multivariable Fine-Gray analysis of factors associated with cardiovascular events (CVEs) in patients with and without beta-blocker therapy.

## Supplementary Table 5. Univariable and Multivariable Cox regression analysis of factors associated with all-cause mortality in patients with and without coronary artery disease.

## Supplementary Table 6. Univariable and Multivariable Fine-Gray analysis of factors associated with cardiovascular events (CVEs) in patients with and without coronary artery disease.

## Supplementary Table 1. Univariable and Multivariable Cox regression analysis of factors associated with all-cause mortality in patients with paroxysmal atrial fibrillation (PAF) and persistent/permanent atrial fibrillation (non-PAF).

|  | **PAF (n: 1,300)** | | | | | | **Non PAF (n: 2,944)** | | | | | |
| --- | --- | --- | --- | --- | --- | --- | --- | --- | --- | --- | --- | --- |
|  | **Univariable** | | | **Multivariable** | | | **Univariable** | | | **Multivariable** | | |
|  | **HR** | **95% CI** | **p-value** | **HR** | **95% CI** | **p-value** | **HR** | **95% CI** | **p-value** | **HR** | **95% CI** | **p-value** |
| **AAD Type** |  |  |  |  |  |  |  |  |  |  |  |  |
| **1c Class** *vs No AAD* | 0.59 | 0.27-1.28 | 0.181 | 0.79 | 0.36-1.76 | 0.566 | 0.22 | 0.06-0.90 | 0.035 | 0.34 | 0.09-1.39 | 0.134 |
| **3 Class AAD** *vs No AAD* | 1.32 | 0.82-2.11 | 0.251 | 1.03 | 0.62-1.72 | 0.903 | 1.09 | 0.77-1.54 | 0.625 | 1.10 | 0.77-1.57 | 0.590 |
| **Age (years)** | 1.10 | 1.05-1.15 | <0.001 | 1.12 | 1.07-1.18 | <0.001 | 1.10 | 1.08-1.13 | <0.001 | 1.12 | 1.08-1.15 | <0.001 |
| **Female** | 0.83 | 0.57-1.21 | 0.327 |  |  |  | 0.74 | 0.61-0.91 | 0.004 | 0.70 | 0.57-0.87 | 0.001 |
| **Hypertension** | 1.01 | 0.61-1.68 | 0.955 |  |  |  | 1.01 | 0.76-1.35 | 0.953 |  |  |  |
| **Diabetes** | 0.95 | 0.56-1.61 | 0.842 |  |  |  | 1.25 | 0.98-1.59 | 0.068 |  |  |  |
| **CAD** | 1.45 | 0.92-2.28 | 0.107 |  |  |  | 1.67 | 1.32-2.11 | <0.001 | 1.48 | 1.14-1.91 | 0.003 |
| **PAD** | 2.47 | 1.43-4.26 | 0.001 | 2.05 | 1.16-3.62 | 0.013 | 1.55 | 1.08-2.23 | 0.018 | 1.34 | 0.93-1.95 | 0.120 |
| **Obesity*** | 1.21 | 0.72-2.03 | 0.469 |  |  |  | 0.86 | 0.64-1.15 | 0.295 |  |  |  |
| **Anaemia** | 1.76 | 1.21-2.56 | 0.003 | 1.39 | 0.94-2.05 | 0.102 | 1.65 | 1.34-2.02 | <0.001 | 1.30 | 1.05-1.61 | 0.016 |
| **Heart failure** | 1.52 | 0.99-2.34 | 0.055 |  |  |  | 1.71 | 1.39-2.10 | <0.001 | 1.15 | 0.91-1.45 | 0.235 |
| **Cancer** | 1.25 | 0.80-1.97 | 0.332 |  |  |  | 1.09 | 0.84-1.42 | 0.524 |  |  |  |
| **Previous stroke/TIA** | 1.39 | 0.91-2.13 | 0.132 |  |  |  | 1.08 | 0.84-1.40 | 0.539 |  |  |  |
| **COPD/OSAS** | 2.34 | 1.51-3.64 | <0.001 | 1.78 | 1.12-2.82 | 0.014 | 2.03 | 1.59-2.58 | <0.001 | 1.58 | 1.22-2.06 | <0.001 |
| **CKD**** | 0.49 | 0.29-0.83 | 0.008 | 0.83 | 0.48-1.42 | 0.494 | 0.57 | 0.43-0.75 | <0.001 | 0.74 | 0.55-1.00 | 0.049 |
| **Alcohol** | 0.84 | 0.34-2.06 | 0.703 |  |  |  | 1.07 | 0.67-1.70 | 0.776 |  |  |  |
| **Dementia** | 2.41 | 1.21-4.79 | 0.012 | 2.75 | 1.37-5.54 | 0.005 | 1.65 | 1.15-2.37 | 0.007 | 1.34 | 0.92-1.95 | 0.127 |
| **Wheelchair users** | 1.41 | 0.66-3.04 | 0.377 |  |  |  | 2.69 | 1.93-3.76 | <0.001 | 1.98 | 1.38-2.84 | <0.001 |
| **Immobilization syndrome** | 0.68 | 0.09-4.89 | 0.702 |  |  |  | 1.53 | 0.73-3.24 | 0.264 |  |  |  |
| **Tendency to fall** | 0.81 | 0.26-2.54 | 0.713 |  |  |  | 1.45 | 0.92-2.27 | 0.107 |  |  |  |
| **Living alone** | 1.25 | 0.58-2.69 | 0.570 |  |  |  | 0.77 | 0.48-1.24 | 0.290 |  |  |  |
| **Social/familial support** | 1.32 | 0.88-1.98 | 0.182 |  |  |  | 1.50 | 1.18-1.90 | <0.001 | 1.34 | 1.04-1.72 | 0.023 |
| **DOAC** *(vs VKA)* | 0.30 | 0.20-0.45 | <0.001 | 0.28 | 0.18-0.43 | <0.001 | 0.45 | 0.36-0.56 | <0.001 | 0.40 | 0.32-0.50 | <0.001 |
| **Aspirin** | 1.45 | 0.88-2.41 | 0.147 |  |  |  | 1.05 | 0.77-1.43 | 0.772 |  |  |  |
| **Lipid lowering therapy** | 0.91 | 0.61-1.35 | 0.634 |  |  |  | 0.72 | 0.57-0.90 | 0.005 | 0.67 | 0.52-0.86 | 0.002 |
| **RAAS inhibitors** | 0.93 | 0.64-1.36 | 0.725 |  |  |  | 0.69 | 0.57-0.85 | <0.001 | 0.75 | 0.60-0.92 | 0.006 |
| **Βeta-blockers** | 1.01 | 0.70-1.47 | 0.947 |  |  |  | 1.03 | 0.84-1.26 | 0.795 |  |  |  |
| **Calcium channel blockers** | 1.20 | 0.79-1.83 | 0.385 |  |  |  | 0.96 | 0.76-1.22 | 0.755 |  |  |  |
| **Diuretics** | 1.24 | 0.85-1.80 | 0.270 |  |  |  | 1.40 | 1.15-1.72 | 0.001 | 1.09 | 0.87-1.36 | 0.464 |
| **Digoxin** | 2.02 | 1.08-3.77 | 0.027 | 2.42 | 1.29-4.56 | 0.006 | 1.11 | 0.84-1.47 | 0.477 |  |  |  |
| **PPI** | 1.56 | 1.07-2.26 | 0.020 | 1.43 | 0.96-2.13 | 0.077 | 1.29 | 1.06-1.58 | 0.013 | 1.01 | 0.81-1.26 | 0.937 |
| **Antipsychotic drugs** | 1.51 | 0.87-2.60 | 0.140 |  |  |  | 1.22 | 0.87-1.69 | 0.250 |  |  |  |
| **Anxiolytic drugs** | 1.84 | 1.11-3.06 | 0.019 | 1.33 | 0.78-2.27 | 0.290 | 1.06 | 0.77-1.46 | 0.721 |  |  |  |
| **Antiepileptic drugs** | 1.30 | 0.48-3.53 | 0.608 |  |  |  | 0.83 | 0.37-1.85 | 0.643 |  |  |  |

*AAD: antiarrhythmics drugs; CAD: coronary artery disease;CI: Confidence Interval; CKD: chronic kidney disease; COPD/OSAS: chronic obstructive pulmonary disease/obstructive sleep apnoea syndrome; DOAC: direct oral anticoagulants; HR: Hazard Ratio; PAD: peripheral artery disease; PPI: proton pump inhibitor; RAAS: renin-angiotensin-aldosterone; VKA: vitamin K antagonist.*

**defined as body mass index≥ 30 Kg/m^2^ **defined as estimated glomerular filtration rate (eGFR)<60 ml/min.*

## Supplementary Table 2. Univariable and Multivariable Fine-Gray analysis of factors associated with cardiovascular events (CVEs) in patients with paroxysmal atrial fibrillation (PAF) and persistent/permanent atrial fibrillation (non-PAF).

|  | **PAF (n: 1,300)** | | | | | | **Non PAF (n: 2,944)** | | | | | |
| --- | --- | --- | --- | --- | --- | --- | --- | --- | --- | --- | --- | --- |
|  | **Univariable** | | | **Multivariable** | | | **Univariable** | | | **Multivariable** | | |
|  | **sHR** | **95% CI** | **p-value** | **sHR** | **95% CI** | **p-value** | **sHR** | **95% CI** | **p-value** | **sHR** | **95% CI** | **p-value** |
| **AAD Type** |  |  |  |  |  |  |  |  |  |  |  |  |
| **1c Class** *(vs No AAD)* | 0.49 | 0.23-1.02 | 0.055 | 0.60 | 0.27-1.31 | 0.200 | 0.52 | 0.22-1.23 | 0.130 | 0.76 | 0.32-1.83 | 0.550 |
| **3 Class AAD (***vs No AAD)* | 1.19 | 0.76-1.86 | 0.450 | 1.03 | 0.63-1.71 | 0.900 | 1.03 | 0.73-1.45 | 0.870 | 1.02 | 0.71-1.45 | 0.930 |
| **Age (years)** | 1.09 | 1.04-1.13 | <0.001 | 1.10 | 1.04-1.15 | <0.001 | 1.10 | 1.08-1.13 | <0.001 | 1.10 | 1.07-1.13 | <0.001 |
| **Female** | 0.73 | 0.52-1.02 | 0.068 |  |  |  | 0.74 | 0.61-0.90 | 0.002 | 0.69 | 0.56-0.85 | <0.001 |
| **Hypertension** | 0.97 | 0.63-1.52 | 0.910 |  |  |  | 0.95 | 0.73-1.25 | 0.730 |  |  |  |
| **Diabetes** | 1.08 | 0.69-1.67 | 0.750 |  |  |  | 1.22 | 0.97-1.53 | 0.093 |  |  |  |
| **CAD** | 1.36 | 0.89-2.07 | 0.160 |  |  |  | 1.66 | 1.32-2.07 | <0.001 | 1.49 | 1.16-1.92 | 0.002 |
| **PAD** | 2.70 | 1.67-4.37 | <0.001 | 2.39 | 1.43-3.98 | <0.001 | 1.60 | 1.13-2.26 | 0.008 | 1.41 | 0.98-2.04 | 0.065 |
| **Obesity*** | 1.07 | 0.64-1.77 | 0.810 |  |  |  | 0.84 | 0.64-1.11 | 0.210 |  |  |  |
| **Anaemia** | 1.60 | 1.13-2.26 | 0.008 | 1.36 | 0.92-2.01 | 0.120 | 1.61 | 1.32-1.96 | <0.001 | 1.29 | 1.05-1.59 | 0.016 |
| **Heart failure** | 1.58 | 1.07-2.35 | 0.022 | 1.15 | 0.75-1.76 | 0.530 | 1.66 | 1.36-2.03 | <0.001 | 1.17 | 0.93-1.47 | 0.180 |
| **Cancer** | 1.40 | 0.93-2.11 | 0.110 |  |  |  | 1.05 | 0.81-1.36 | 0.720 |  |  |  |
| **Previous stroke/TIA** | 1.44 | 0.98-2.14 | 0.065 |  |  |  | 1.17 | 0.92-1.49 | 0.200 |  |  |  |
| **COPD/OSAS** | 2.19 | 1.44-3.32 | <0.001 | 1.76 | 1.09-2.84 | 0.020 | 1.97 | 1.55-2.51 | <0.001 | 1.58 | 1.22-2.06 | <0.001 |
| **CKD**** | 0.52 | 0.33-0.81 | 0.004 | 0.74 | 0.46-1.19 | 0.210 | 0.54 | 0.42-0.71 | <0.001 | 0.66 | 0.50-0.89 | 0.005 |
| **Alcohol** | 1.64 | 0.90-2.99 | 0.110 |  |  |  | 1.14 | 0.73-1.77 | 0.570 |  |  |  |
| **Dementia** | 2.02 | 0.98-4.14 | 0.055 |  |  |  | 1.65 | 1.15-2.37 | 0.007 | 1.33 | 0.90-1.96 | 0.160 |
| **Wheelchair users** | 1.55 | 0.78-3.10 | 0.210 |  |  |  | 2.59 | 1.90-3.55 | <0.001 | 1.88 | 1.31-2.69 | <0.001 |
| **Immobilization syndrome** | 0.58 | 0.08-4.28 | 0.600 |  |  |  | 1.60 | 0.80-3.21 | 0.180 |  |  |  |
| **Tendency to fall** | 1.39 | 0.64-3.02 | 0.410 |  |  |  | 1.38 | 0.86-2.21 | 0.180 |  |  |  |
| **Living alone** | 1.04 | 0.49-2.20 | 0.920 |  |  |  | 0.83 | 0.54-1.28 | 0.400 |  |  |  |
| **Social/familial support** | 1.39 | 0.94-2.06 | 0.098 |  |  |  | 1.46 | 1.17-1.82 | <0.001 | 1.35 | 1.07-1.71 | 0.013 |
| **DOAC** *(vs VKA)* | 0.44 | 0.31-0.62 | <0.001 | 0.44 | 0.30-0.65 | <0.001 | 0.54 | 0.44-0.67 | <0.001 | 0.49 | 0.39-0.62 | <0.001 |
| **Concomitant Aspirin** | 1.59 | 1.02-2.48 | 0.039 | 1.15 | 0.70-1.89 | 0.580 | 1.12 | 0.83-1.51 | 0.470 |  |  |  |
| **Lipid lowering therapy** | 0.98 | 0.69-1.40 | 0.910 |  |  |  | 0.73 | 0.59-0.91 | 0.005 | 0.68 | 0.54-0.87 | 0.002 |
| **RAAS inhibitors** | 0.95 | 0.67-1.34 | 0.760 |  |  |  | 0.69 | 0.57-0.84 | <0.001 | 0.73 | 0.60-0.90 | 0.003 |
| **Beta blockers** | 0.97 | 0.69-1.37 | 0.850 |  |  |  | 0.96 | 0.79-1.16 | 0.650 |  |  |  |
| **Calcium channel blockers** | 1.14 | 0.77-1.69 | 0.500 |  |  |  | 0.93 | 0.74-1.18 | 0.560 |  |  |  |
| **Diuretics** | 1.13 | 0.80-1.59 | 0.500 |  |  |  | 1.32 | 1.09-1.61 | 0.004 | 1.04 | 0.84-1.29 | 0.710 |
| **Digoxin** | 2.00 | 1.06-3.78 | 0.033 | 2.38 | 1.26-4.48 | 0.007 | 1.08 | 0.81-1.42 | 0.610 |  |  |  |
| **PPI** | 1.41 | 1.00-2.00 | 0.052 |  |  |  | 1.23 | 1.01, 1.49 | 0.043 | 0.97 | 0.78-1.21 | 0.790 |
| **Antipsychotic drugs** | 1.73 | 1.06-2.83 | 0.028 | 1.95 | 1.19-3.19 | 0.008 | 1.13 | 0.82, 1.57 | 0.450 |  |  |  |
| **Anxiolytic drugs** | 1.81 | 1.15-2.86 | 0.011 | 1.28 | 0.77-2.14 | 0.340 | 1.03 | 0.76, 1.41 | 0.840 |  |  |  |
| **Antiepileptic drugs** | 1.10 | 0.37-3.22 | 0.860 |  |  |  | 0.89 | 0.42, 1.86 | 0.750 |  |  |  |

*AAD: antiarrhythmics drugs; CAD: coronary artery disease; CI: Confidence Interval; CKD: chronic kidney disease; COPD/OSAS: chronic obstructive pulmonary disease/obstructive sleep apnoea syndrome; DOAC: direct oral anticoagulants; PAD: peripheral artery disease; PPI: proton pump inhibitor; RAAS: renin-angiotensin-aldosterone; sHR: subdistribution Hazard Ratio; VKA: vitamin K antagonist.*

**defined as body mass index≥ 30 Kg/m^2^ **defined as estimated glomerular filtration rate (eGFR)<60 ml/min.*

## Supplementary Table 3. Univariable and Multivariable Cox regression analysis of factors associated with all-cause mortality in patients with and without beta-blocker therapy.

| **Variable** | **BB users (n: 1,932)** | | | | | | | **Non-BB users (n: 2,312)** | | | | | | | |  |
| --- | --- | --- | --- | --- | --- | --- | --- | --- | --- | --- | --- | --- | --- | --- | --- | --- |
|  | **Univariable** | | | **Multivariable** | | | | **Univariable** | | | | **Multivariable** | | | |  |
|  | **HR** | **95% CI** | **p-value** | | **HR** | **95% CI** | **p-value** | | **HR** | **95% CI** | **p-value** | | **HR** | **95% CI** | **p-value** | |
| **AAD Type** |  |  |  | |  |  |  | |  |  |  | |  |  |  | |
| **1c Class** *(vs No AAD)* | 0.51 | 0.19, 1.38 | 0.186 | | 0.81 | 0.30, 2.21 | 0.686 | | 0.28 | 0.12, 0.69 | 0.006 | | 0.43 | 0.17, 1.08 | 0.074 | |
| **3 Class AAD** (*vs No AAD)* | 1.23 | 0.83, 1.82 | 0.312 | | 1.2 | 0.79, 1.80 | 0.393 | | 0.93 | 0.63, 1.38 | 0.728 | | 1.05 | 0.70, 1.60 | 0.803 | |
| **Age (years)** | 1.1 | 1.07, 1.14 | <0.001 | | 1.12 | 1.08, 1.17 | <0.001 | | 1.11 | 1.08, 1.15 | <0.001 | | 1.12 | 1.08, 1.16 | <0.001 | |
| **Female** | 0.67 | 0.52, 0.86 | 0.002 | | 0.61 | 0.47, 0.80 | <0.001 | | 0.82 | 0.64, 1.06 | 0.131 | |  |  |  | |
| **PAF** | 0.67 | 0.49, 0.91 | 0.010 | | 0.71 | 0.52, 0.97 | 0.033 | | 0.66 | 0.49, 0.89 | 0.006 | | 0.89 | 0.65, 1.21 | 0.452 | |
| **Hypertension** | 0.87 | 0.60, 1.26 | 0.458 | |  |  |  | | 1.14 | 0.81, 1.60 | 0.453 | |  |  |  | |
| **Diabetes** | 1.23 | 0.91, 1.67 | 0.176 | |  |  |  | | 1.19 | 0.87, 1.63 | 0.284 | |  |  |  | |
| **CAD** | 1.53 | 1.16, 2.03 | 0.003 | | 1.29 | 0.94, 1.78 | 0.115 | | 1.71 | 1.25, 2.34 | <0.001 | | 1.26 | 0.91, 1.76 | 0.164 | |
| **PAD** | 1.66 | 1.10, 2.51 | 0.016 | | 1.37 | 0.89, 2.11 | 0.150 | | 1.89 | 1.22, 2.93 | 0.005 | | 1.73 | 1.09, 2.73 | 0.019 | |
| **Obesity*** | 0.88 | 0.62, 1.25 | 0.467 | |  |  |  | | 1.02 | 0.70, 1.47 | 0.932 | |  |  |  | |
| **Anaemia** | 1.64 | 1.27, 2.10 | <0.001 | | 1.23 | 0.94, 1.60 | 0.134 | | 1.68 | 1.30, 2.16 | <0.001 | | 1.35 | 1.03, 1.76 | 0.029 | |
| **Heart failure** | 1.37 | 1.06, 1.78 | 0.016 | | 0.99 | 0.74, 1.32 | 0.932 | | 2.30 | 1.75, 3.01 | <0.001 | | 1.33 | 0.98, 1.82 | 0.071 | |
| **Cancer** | 0.75 | 0.52, 1.08 | 0.119 | |  |  |  | | 1.56 | 1.16, 2.10 | 0.003 | | 1.47 | 1.08, 2.01 | 0.015 | |
| **Previous stroke/TIA** | 1.14 | 0.84, 1.55 | 0.403 | |  |  |  | | 1.12 | 0.82, 1.53 | 0.480 | |  |  |  | |
| **COPD/OSAS** | 1.89 | 1.39, 2.56 | <0.001 | | 1.54 | 1.11, 2.13 | 0.010 | | 2.4 | 1.78, 3.24 | <0.001 | | 1.85 | 1.34, 2.55 | <0.001 | |
| **CKD**** | 0.48 | 0.33, 0.70 | <0.001 | | 0.65 | 0.44, 0.96 | 0.029 | | 0.6 | 0.43, 0.83 | 0.002 | | 0.88 | 0.62, 1.25 | 0.485 | |
| **Alcohol** | 0.95 | 0.56, 1.64 | 0.866 | |  |  |  | | 1.03 | 0.55, 1.95 | 0.923 | |  |  |  | |
| **Dementia** | 2.11 | 1.39, 3.22 | <0.001 | | 1.63 | 1.03, 2.58 | 0.038 | | 1.55 | 0.94, 2.53 | 0.084 | |  |  |  | |
| **Wheelchair users** | 2.2 | 1.43, 3.39 | <0.001 | | 1.9 | 1.19, 3.02 | 0.007 | | 2.44 | 1.59, 3.76 | <0.001 | | 1.24 | 0.75, 2.05 | 0.403 | |
| **Immobilization syndrome** | 0.35 | 0.05, 2.48 | 0.293 | |  |  |  | | 2.17 | 1.02, 4.60 | 0.044 | | 1.21 | 0.54, 2.71 | 0.646 | |
| **Tendency to fall** | 1.3 | 0.75, 2.28 | 0.353 | |  |  |  | | 1.36 | 0.72, 2.57 | 0.340 | |  |  |  | |
| **Living alone** | 0.91 | 0.54, 1.53 | 0.709 | |  |  |  | | 0.76 | 0.40, 1.44 | 0.397 | |  |  |  | |
| **Social/familial support** | 1.46 | 1.09, 1.97 | 0.012 | | 1.13 | 0.82, 1.54 | 0.463 | | 1.52 | 1.15, 2.02 | 0.004 | | 1.40 | 1.03, 1.89 | 0.031 | |
| **DOAC** *(vs VKA)* | 0.37 | 0.28, 0.48 | <0.001 | | 0.31 | 0.23, 0.42 | <0.001 | | 0.45 | 0.34, 0.59 | <0.001 | | 0.44 | 0.33, 0.59 | <0.001 | |
| **Concomitant Aspirin** | 0.97 | 0.66, 1.43 | 0.887 | |  |  |  | | 1.34 | 0.93, 1.94 | 0.118 | |  |  |  | |
| **Lipid lowering therapy** | 0.74 | 0.56, 0.97 | 0.030 | | 0.75 | 0.55, 1.01 | 0.062 | | 0.76 | 0.56, 1.02 | 0.067 | |  |  |  | |
| **RAAS inhibitors** | 0.72 | 0.56, 0.92 | 0.008 | | 0.75 | 0.58, 0.97 | 0.026 | | 0.74 | 0.57, 0.95 | 0.020 | | 0.82 | 0.63, 1.08 | 0.153 | |
| **Calcium channel blockers** | 0.81 | 0.59, 1.13 | 0.215 | |  |  |  | | 1.24 | 0.94, 1.63 | 0.125 | |  |  |  | |
| **Diuretics** | 1.40 | 1.09, 1.81 | 0.009 | | 0.98 | 0.73, 1.30 | 0.871 | | 1.43 | 1.10, 1.84 | 0.007 | | 1.03 | 0.78, 1.37 | 0.832 | |
| **Digoxin** | 0.85 | 0.58, 1.24 | 0.402 | |  |  |  | | 2.03 | 1.43, 2.87 | <0.001 | | 1.59 | 1.10, 2.30 | 0.014 | |
| **PPI** | 1.30 | 1.01, 1.66 | 0.039 | | 1.07 | 0.82, 1.39 | 0.637 | | 1.45 | 1.12, 1.88 | 0.005 | | 1.13 | 0.84, 1.51 | 0.428 | |
| **Antipsychotic drugs** | 1.5 | 1.04, 2.16 | 0.030 | | 1.72 | 1.17, 2.52 | 0.006 | | 1.01 | 0.65, 1.59 | 0.955 | |  |  |  | |
| **Anxiolytic drugs** | 1.00 | 0.69, 1.47 | 0.985 | |  |  |  | | 1.52 | 1.04, 2.22 | 0.029 | | 1.34 | 0.91, 1.98 | 0.138 | |
| **Antiepileptic drugs** | 0.71 | 0.26, 1.91 | 0.497 | |  |  |  | | 1.11 | 0.49, 2.51 | 0.795 | |  |  |  | |

*AAD: antiarrhythmics drugs; BB: Beta blocker; CAD: coronary artery disease; CI: Confidence Interval; CKD: chronic kidney disease; COPD/OSAS: chronic obstructive pulmonary disease/obstructive sleep apnoea syndrome; DOAC: direct oral anticoagulants; HR: Hazard Ratio; PAD: peripheral artery disease; PAF: Paroxysmal Atrial Fibrillation; PPI: proton pump inhibitor; RAAS: renin-angiotensin-aldosterone; VKA: vitamin K antagonist.*

**defined as body mass index≥ 30 Kg/m^2^ **defined as estimated glomerular filtration rate (eGFR)<60 ml/min.*

## Supplementary Table 4. Univariable and Multivariable Fine-Gray analysis of factors associated with cardiovascular events (CVEs) in patients with and without beta-blocker therapy.

| **Variable** | **BB users (n: 1,932)** | | | | | | **Non-BB users (n: 2,312)** | | | | | |
| --- | --- | --- | --- | --- | --- | --- | --- | --- | --- | --- | --- | --- |
|  | **Univariable** | | | **Multivariable** | | | **Univariable** | | | **Multivariable** | | |
|  | **sHR** | **95% CI** | **p-value** | **sHR** | **95% CI** | **p-value** | **sHR** | **95% CI** | **p-value** | **sHR** | **95% CI** | **p-value** |
| **AAD Type** |  |  |  |  |  |  |  |  |  |  |  |  |
| **1c Class** *(vs No AAD)* | 0.59 | 0.24, 1.44 | 0.250 | 0.90 | 0.37, 2.18 | 0.820 | 0.35 | 0.17, 0.71 | 0.004 | 0.49 | 0.22, 1.08 | 0.075 |
| **3 Class AAD** (*vs No AAD)* | 1.18 | 0.80, 1.75 | 0.410 | 1.09 | 0.72, 1.65 | 0.670 | 0.87 | 0.59, 1.28 | 0.470 | 0.97 | 0.66, 1.44 | 0.890 |
| **Age (years)** | 1.10 | 1.07, 1.13 | <0.001 | 1.11 | 1.08, 1.15 | <0.001 | 1.10 | 1.07, 1.13 | <0.001 | 1.10 | 1.07, 1.13 | <0.001 |
| **Female** | 0.67 | 0.53, 0.86 | 0.001 | 0.61 | 0.47, 0.80 | <0.001 | 0.78 | 0.62, 1.0 | 0.045 | 0.72 | 0.55, 0.94 | 0.017 |
| **PAF** | 0.73 | 0.55, 0.97 | 0.029 | 0.77 | 0.57, 1.03 | 0.077 | 0.70 | 0.53, 0.93 | 0.012 | 0.92 | 0.69, 1.23 | 0.580 |
| **Hypertension** | 0.86 | 0.60, 1.22 | 0.390 |  |  |  | 1.05 | 0.77, 1.43 | 0.740 |  |  |  |
| **Diabetes** | 1.20 | 0.90, 1.61 | 0.220 |  |  |  | 1.21 | 0.91, 1.61 | 0.190 |  |  |  |
| **CAD** | 1.56 | 1.19, 2.04 | 0.001 | 1.23 | 0.92, 1.65 | 0.160 | 1.62 | 1.21, 2.16 | 0.001 | 1.15 | 0.84, 1.59 | 0.390 |
| **PAD** | 1.80 | 1.21, 2.68 | 0.004 | 1.42 | 0.92, 2.19 | 0.110 | 1.95 | 1.32, 2.86 | <0.001 | 1.78 | 1.20, 2.65 | 0.004 |
| **Obesity*** | 0.83 | 0.59, 1.16 | 0.280 |  |  |  | 0.99 | 0.70, 1.40 | 0.940 |  |  |  |
| **Anaemia** | 1.54 | 1.21, 1.97 | <0.001 | 1.18 | 0.91, 1.53 | 0.220 | 1.63 | 1.29, 2.08 | <0.001 | 1.29 | 1.00, 1.67 | 0.051 |
| **Heart failure** | 1.40 | 1.09, 1.79 | 0.009 | 1.04 | 0.78, 1.38 | 0.790 | 2.18 | 1.69, 2.79 | <0.001 | 1.41 | 1.03, 1.92 | 0.031 |
| **Cancer** | 0.75 | 0.53, 1.07 | 0.120 |  |  |  | 1.54 | 1.16, 2.05 | 0.003 | 1.45 | 1.07, 1.98 | 0.017 |
| **Previous stroke/TIA** | 1.22 | 0.91, 1.63 | 0.180 |  |  |  | 1.21 | 0.90, 1.61 | 0.200 |  |  |  |
| **COPD/OSAS** | 1.84 | 1.37, 2.47 | <0.001 | 1.54 | 1.12, 2.12 | 0.008 | 2.28 | 1.70, 3.06 | <0.001 | 1.76 | 1.26, 2.45 | <0.001 |
| **CKD**** | 0.49 | 0.35, 0.69 | <0.001 | 0.63 | 0.44, 0.91 | 0.015 | 0.56 | 0.41, 0.76 | <0.001 | 0.68 | 0.48, 0.96 | 0.030 |
| **Alcohol** | 1.37 | 0.88, 2.12 | 0.170 |  |  |  | 1.08 | 0.59, 1.97 | 0.810 |  |  |  |
| **Dementia** | 2.04 | 1.33, 3.12 | 0.001 | 1.57 | 0.96, 2.55 | 0.071 | 1.50 | 0.91, 2.47 | 0.110 |  |  |  |
| **Wheelchair users** | 2.24 | 1.49, 3.37 | <0.001 | 1.86 | 1.19, 2.90 | 0.006 | 2.29 | 1.51, 3.48 | <0.001 | 1.20 | 0.72, 1.98 | 0.490 |
| **Immobilization syndrome** | 0.65 | 0.16, 2.58 | 0.540 |  |  |  | 1.88 | 0.89, 3.99 | 0.098 |  |  |  |
| **Tendency to fall** | 1.39 | 0.80, 2.41 | 0.240 |  |  |  | 1.42 | 0.80, 2.54 | 0.240 |  |  |  |
| **Living alone** | 0.96 | 0.59, 1.55 | 0.860 |  |  |  | 0.73 | 0.39, 1.35 | 0.320 |  |  |  |
| **Social/familial support** | 1.43 | 1.07, 1.89 | 0.014 | 1.19 | 0.89, 1.58 | 0.240 | 1.54 | 1.19, 2.00 | 0.001 | 1.44 | 1.08, 1.92 | 0.012 |
| **DOAC** *(vs VKA)* | 0.44 | 0.34, 0.58 | <0.001 | 0.38 | 0.29, 0.49 | <0.001 | 0.58 | 0.45, 0.75 | <0.001 | 0.61 | 0.46, 0.81 | <0.001 |
| **Concomitant Aspirin** | 1.07 | 0.74, 1.53 | 0.720 |  |  |  | 1.42 | 1.02, 1.98 | 0.036 | 1.10 | 0.75, 1.61 | 0.640 |
| **Lipid lowering therapy** | 0.78 | 0.60, 1.01 | 0.059 |  |  |  | 0.79 | 0.61, 1.03 | 0.083 |  |  |  |
| **RAAS inhibitors** | 0.72 | 0.57, 0.92 | 0.007 | 0.72 | 0.56, 0.92 | 0.009 | 0.75 | 0.59, 0.95 | 0.018 | 0.78 | 0.60, 1.01 | 0.059 |
| **Calcium channel blockers** | 0.80 | 0.59, 1.10 | 0.180 |  |  |  | 1.16 | 0.89, 1.50 | 0.280 |  |  |  |
| **Diuretics** | 1.32 | 1.03, 1.68 | 0.026 | 0.93 | 0.71, 1.23 | 0.620 | 1.34 | 1.06, 1.70 | 0.016 | 0.97 | 0.74, 1.27 | 0.820 |
| **Digoxin** | 0.81 | 0.55, 1.19 | 0.280 |  |  |  | 1.99 | 1.42, 2.78 | <0.001 | 1.63 | 1.13, 2.35 | 0.009 |
| **PPI** | 1.21 | 0.96, 1.54 | 0.110 |  |  |  | 1.39 | 1.08, 1.77 | 0.009 | 1.09 | 0.82, 1.45 | 0.540 |
| **Antipsychotic drugs** | 1.53 | 1.07, 2.18 | 0.021 | 1.71 | 1.17, 2.48 | 0.005 | 1.01 | 0.66, 1.53 | 0.970 |  |  |  |
| **Anxiolytic drugs** | 0.96 | 0.66, 1.39 | 0.810 |  |  |  | 1.55 | 1.08, 2.22 | 0.017 | 1.48 | 1.00, 2.19 | 0.050 |
| **Antiepileptic drugs** | 0.67 | 0.25, 1.79 | 0.420 |  |  |  | 1.11 | 0.50, 2.50 | 0.790 |  |  |  |

*AAD: antiarrhythmics drugs; BB: Beta blocker; CAD: coronary artery disease; CI: Confidence Interval; CKD: chronic kidney disease; COPD/OSAS: chronic obstructive pulmonary disease/obstructive sleep apnoea syndrome; DOAC: direct oral anticoagulants; PAD: peripheral artery disease; PAF: Paroxysmal Atrial Fibrillation; PPI: proton pump inhibitor; RAAS: renin-angiotensin-aldosterone; sHR: subdistribution Hazard Ratio; VKA: vitamin K antagonist.*

**defined as body mass index≥ 30 Kg/m^2^ **defined as estimated glomerular filtration rate (eGFR)<60 ml/min.*

## Supplementary Table 5. Univariable and Multivariable Cox regression analysis of factors associated with all-cause mortality in patients with and without coronary artery disease.

| **Variable** | **CAD (n: 719)** | | | | | | **No CAD (n: 3,525)** | | | | | |
| --- | --- | --- | --- | --- | --- | --- | --- | --- | --- | --- | --- | --- |
|  | **Univariable** | | | **Multivariable** | | | **Univariable** | | | **Multivariable** | | |
|  | **HR** | **95% CI** | **p-value** | **HR** | **95% CI** | **p-value** | **HR** | **95% CI** | **p-value** | **HR** | **95% CI** | **p-value** |
| **AAD Type** |  |  |  |  |  |  |  |  |  |  |  |  |
| **1c Class** *(vs No AAD)^†^* | NA | NA, NA | NA | NA | NA, NA | NA | 0.45 | 0.23, 0.87 | 0.017 | 0.65 | 0.33, 1.27 | 0.205 |
| **3 Class AAD (***vs No AAD)* | 0.86 | 0.52, 1.45 | 0.582 | 1.09 | 0.63, 1.87 | 0.762 | 1.12 | 0.81, 1.56 | 0.485 | 1.14 | 0.81, 1.59 | 0.457 |
| **Age (years)** | 1.10 | 1.05, 1.15 | <0.001 | 1.11 | 1.06, 1.17 | <0.001 | 1.11 | 1.08, 1.14 | <0.001 | 1.12 | 1.09, 1.16 | <0.001 |
| **Female** | 0.56 | 0.38, 0.85 | 0.006 | 0.67 | 0.44, 1.02 | 0.06 | 0.86 | 0.70, 1.06 | 0.161 |  |  |  |
| **Hypertension** | 0.85 | 0.48, 1.52 | 0.586 |  |  |  | 1.02 | 0.77, 1.35 | 0.888 |  |  |  |
| **Diabetes** | 0.89 | 0.57, 1.37 | 0.586 |  |  |  | 1.29 | 1.00, 1.66 | 0.047 | 1.36 | 1.05, 1.77 | 0.02 |
| **PAF** | 0.59 | 0.38, 0.93 | 0.023 | 0.72 | 0.45, 1.14 | 0.159 | 0.70 | 0.55, 0.89 | 0.003 | 0.84 | 0.66, 1.08 | 0.182 |
| **PAD** | 1.77 | 1.12, 2.79 | 0.015 | 1.98 | 1.22, 3.21 | 0.006 | 1.47 | 0.97, 2.22 | 0.069 |  |  |  |
| **Obesity*** | 1.05 | 0.62, 1.78 | 0.857 |  |  |  | 0.91 | 0.68, 1.22 | 0.524 |  |  |  |
| **Anaemia** | 2.06 | 1.43, 2.98 | <0.001 | 1.79 | 1.20, 2.66 | 0.004 | 1.50 | 1.22, 1.85 | <0.001 | 1.18 | 0.95, 1.47 | 0.123 |
| **Heart failure** | 1.27 | 0.88, 1.83 | 0.203 |  |  |  | 1.78 | 1.43, 2.21 | <0.001 | 1.23 | 0.96, 1.57 | 0.099 |
| **Cancer** | 1.28 | 0.83, 1.97 | 0.258 |  |  |  | 1.03 | 0.79, 1.35 | 0.817 |  |  |  |
| **Previous stroke/TIA** | 1.37 | 0.90, 2.08 | 0.138 |  |  |  | 1.06 | 0.82, 1.38 | 0.637 |  |  |  |
| **COPD/OSAS** | 1.63 | 1.06, 2.50 | 0.027 | 1.28 | 0.83, 1.99 | 0.265 | 2.22 | 1.74, 2.83 | <0.001 | 1.88 | 1.45, 2.43 | <0.001 |
| **CKD**** | 0.62 | 0.37, 1.06 | 0.079 |  |  |  | 0.54 | 0.41, 0.71 | <0.001 | 0.79 | 0.59, 1.06 | 0.122 |
| **Alcohol** | 1.16 | 0.54, 2.50 | 0.698 |  |  |  | 0.94 | 0.58, 1.53 | 0.805 |  |  |  |
| **Dementia** | 1.3 | 0.63, 2.67 | 0.477 |  |  |  | 1.96 | 1.37, 2.81 | <0.001 | 1.61 | 1.11, 2.35 | 0.012 |
| **Wheelchair users** | 1.85 | 0.90, 3.81 | 0.096 |  |  |  | 2.54 | 1.81, 3.55 | <0.001 | 1.77 | 1.22, 2.56 | 0.003 |
| **Immobilization syndrome** | 4.03 | 1.27, 12.7 | 0.018 | 2.64 | 0.79, 8.88 | 0.116 | 0.99 | 0.41, 2.40 | 0.984 |  |  |  |
| **Tendency to fall** | 1.24 | 0.45, 3.36 | 0.677 |  |  |  | 1.37 | 0.87, 2.18 | 0.178 |  |  |  |
| **Living alone** | 1.05 | 0.46, 2.40 | 0.904 |  |  |  | 0.83 | 0.52, 1.32 | 0.429 |  |  |  |
| **Social/familial support** | 1.09 | 0.73, 1.63 | 0.667 |  |  |  | 1.60 | 1.27, 2.03 | <0.001 | 1.40 | 1.09, 1.80 | 0.009 |
| **DOAC** *(vs VKA)* | 0.35 | 0.23, 0.55 | <0.001 | 0.34 | 0.21, 0.53 | <0.001 | 0.43 | 0.34, 0.53 | <0.001 | 0.36 | 0.29, 0.45 | <0.001 |
| **Concomitant Aspirin** | 0.78 | 0.52, 1.17 | 0.228 |  |  |  | 1.11 | 0.76, 1.63 | 0.576 |  |  |  |
| **Lipid lowering therapy** | 0.69 | 0.48, 1.00 | 0.051 |  |  |  | 0.58 | 0.44, 0.76 | <0.001 | 0.65 | 0.49, 0.85 | 0.002 |
| **RAAS inhibitors** | 0.63 | 0.44, 0.91 | 0.014 | 0.69 | 0.46, 1.01 | 0.058 | 0.74 | 0.60, 0.90 | 0.003 | 0.81 | 0.66, 1.0 | 0.044 |
| **Beta blockers** | 0.95 | 0.66, 1.38 | 0.794 |  |  |  | 1.03 | 0.84, 1.26 | 0.800 |  |  |  |
| **Calcium channel blockers** | 1.15 | 0.77, 1.72 | 0.507 |  |  |  | 0.97 | 0.76, 1.23 | 0.782 |  |  |  |
| **Diuretics** | 1.57 | 1.06, 2.30 | 0.023 | 1.20 | 0.79, 1.81 | 0.394 | 1.29 | 1.05, 1.58 | 0.013 | 0.94 | 0.75, 1.18 | 0.576 |
| **Digoxin** | 1.34 | 0.77, 2.35 | 0.306 |  |  |  | 1.28 | 0.96, 1.71 | 0.093 |  |  |  |
| **PPI** | 1.22 | 0.84, 1.75 | 0.293 |  |  |  | 1.33 | 1.09, 1.64 | 0.006 | 1.15 | 0.92, 1.44 | 0.215 |
| **Antipsychotic drugs** | 1.13 | 0.57, 2.24 | 0.716 |  |  |  | 1.34 | 0.98, 1.83 | 0.066 |  |  |  |
| **Anxiolytic drugs** | 1.65 | 0.99, 2.76 | 0.057 |  |  |  | 1.12 | 0.82, 1.53 | 0.479 |  |  |  |
| **Antiepileptic drugs** | 1.22 | 0.30, 4.94 | 0.782 |  |  |  | 0.93 | 0.46, 1.87 | 0.829 |  |  |  |

*AAD: antiarrhythmics drugs;CAD: coronary artery disease; CI: Confidence Interval; CKD: chronic kidney disease; COPD/OSAS: chronic obstructive pulmonary disease/obstructive sleep apnoea syndrome; DOAC: direct oral anticoagulants; HR: Hazard Ratio; PAD: peripheral artery disease; PAF: Paroxysmal Atrial Fibrillation; PPI: proton pump inhibitor; RAAS: renin-angiotensin-aldosterone; VKA: vitamin K antagonist.*

*†No deaths occurred in CAD group *defined as body mass index≥ 30 Kg/m^2^ **defined as estimated glomerular filtration rate (eGFR)<60 ml/min.*

## Supplementary Table 6. Univariable and Multivariable Fine-Gray analysis of factors associated with cardiovascular events (CVEs) in patients with and without coronary artery disease.

|  | **CAD (n: 719)** | | | | | | **No CAD (n: 3,525)** | | | | | |
| --- | --- | --- | --- | --- | --- | --- | --- | --- | --- | --- | --- | --- |
|  | **Univariable** | | | **Multivariable** | | | **Univariable** | | | **Multivariable** | | |
|  | **sHR** | **95% CI** | **p-value** | **sHR** | **95% CI** | **p-value** | **sHR** | **95% CI** | **p-value** | **sHR** | **95% CI** | **p-value** |
| **AAD Type** |  |  |  |  |  |  |  |  |  |  |  |  |
| **1c Class** *(vs No AAD)^†^* | NA | NA, NA | NA | NA | NA, NA | NA | 0.53 | 0.30, 0.93 | 0.028 | 0.70 | 0.39, 1.28 | 0.250 |
| **3 Class AAD (***vs No AAD)* | 0.82 | 0.49, 1.37 | 0.45 | 0.98 | 0.57, 1.67 | 0.94 | 1.06 | 0.77, 1.46 | 0.74 | 1.03 | 0.74, 1.43 | 0.880 |
| **Age (years)** | 1.10 | 1.06, 1.15 | <0.001 | 1.12 | 1.06, 1.17 | <0.001 | 1.10 | 1.07, 1.12 | <0.001 | 1.10 | 1.07, 1.13 | <0.001 |
| **Female** | 0.54 | 0.37, 0.80 | 0.002 | 0.63 | 0.43, 0.93 | 0.020 | 0.84 | 0.69, 1.02 | 0.073 |  |  |  |
| **Hypertension** | 0.86 | 0.51, 1.46 | 0.57 |  |  |  | 0.95 | 0.74, 1.23 | 0.7 |  |  |  |
| **Diabetes** | 0.94 | 0.63, 1.41 | 0.78 |  |  |  | 1.25 | 0.99, 1.59 | 0.06 |  |  |  |
| **PAF** | 0.61 | 0.40, 0.93 | 0.022 | 0.72 | 0.47, 1.09 | 0.120 | 0.76 | 0.61, 0.95 | 0.016 | 0.91 | 0.72, 1.14 | 0.400 |
| **PAD** | 1.67 | 1.06, 2.63 | 0.028 | 1.68 | 1.06, 2.67 | 0.027 | 1.72 | 1.19, 2.48 | 0.004 | 1.60 | 1.07, 2.41 | 0.023 |
| **Obesity*** | 1.08 | 0.66, 1.77 | 0.74 |  |  |  | 0.85 | 0.64, 1.13 | 0.26 |  |  |  |
| **Anaemia** | 2.00 | 1.40, 2.84 | <0.001 | 1.83 | 1.26, 2.67 | 0.002 | 1.44 | 1.18, 1.76 | <0.001 | 1.17 | 0.94, 1.44 | 0.150 |
| **Heart failure** | 1.28 | 0.90, 1.82 | 0.16 |  |  |  | 1.73 | 1.41, 2.13 | <0.001 | 1.23 | 0.97, 1.55 | 0.081 |
| **Cancer** | 1.30 | 0.86, 1.96 | 0.21 |  |  |  | 1.04 | 0.80, 1.35 | 0.77 |  |  |  |
| **Previous stroke/TIA** | 1.32 | 0.89, 1.96 | 0.17 |  |  |  | 1.19 | 0.94, 1.51 | 0.15 |  |  |  |
| **COPD/OSAS** | 1.68 | 1.10, 2.55 | 0.016 | 1.40 | 0.92, 2.12 | 0.120 | 2.10 | 1.65, 2.67 | <0.001 | 1.77 | 1.37, 2.29 | <0.001 |
| **CKD**** | 0.65 | 0.41, 1.03 | 0.067 |  |  |  | 0.52 | 0.40, 0.67 | <0.001 | 0.71 | 0.54, 0.94 | 0.015 |
| **Alcohol** | 1.39 | 0.73, 2.65 | 0.32 |  |  |  | 1.20 | 0.79, 1.83 | 0.39 |  |  |  |
| **Dementia** | 1.17 | 0.55, 2.48 | 0.68 |  |  |  | 1.92 | 1.34, 2.75 | <0.001 | 1.46 | 0.98, 2.18 | 0.066 |
| **Wheelchair users** | 1.69 | 0.84, 3.41 | 0.14 |  |  |  | 2.51 | 1.82, 3.46 | <0.001 | 1.77 | 1.23, 2.54 | 0.002 |
| **Immobilization syndrome** | 3.78 | 1.43, 9.95 | 0.007 | 2.48 | 0.98, 6.26 | 0.055 | 1.06 | 0.48, 2.36 | 0.88 |  |  |  |
| **Tendency to fall** | 1.11 | 0.40, 3.05 | 0.84 |  |  |  | 1.48 | 0.95, 2.30 | 0.081 |  |  |  |
| **Living alone** | 0.93 | 0.41, 2.10 | 0.86 |  |  |  | 0.86 | 0.56, 1.33 | 0.51 |  |  |  |
| **Social/familial support** | 1.10 | 0.75, 1.63 | 0.62 |  |  |  | 1.57 | 1.27, 1.96 | <0.001 | 1.43 | 1.13, 1.81 | 0.003 |
| **DOAC** *(vs VKA)* | 0.45 | 0.30, 0.67 | <0.001 | 0.42 | 0.28, 0.65 | <0.001 | 0.54 | 0.44, 0.66 | <0.001 | 0.48 | 0.39, 0.60 | <0.001 |
| **Concomitant Aspirin** | 0.90 | 0.62, 1.30 | 0.58 |  |  |  | 1.17 | 0.82, 1.67 | 0.39 |  |  |  |
| **Lipid lowering therapy** | 0.70 | 0.49, 1.00 | 0.051 |  |  |  | 0.63 | 0.49, 0.80 | <0.001 | 0.70 | 0.54, 0.90 | 0.006 |
| **RAAS inhibitors** | 0.71 | 0.50, 1.01 | 0.057 |  |  |  | 0.72 | 0.59, 0.87 | <0.001 | 0.77 | 0.63, 0.94 | 0.011 |
| **Beta blockers** | 0.94 | 0.66, 1.32 | 0.71 |  |  |  | 0.94 | 0.78, 1.15 | 0.56 |  |  |  |
| **Calcium channel blockers** | 1.05 | 0.71, 1.57 | 0.79 |  |  |  | 0.95 | 0.76, 1.20 | 0.68 |  |  |  |
| **Diuretics** | 1.49 | 1.04, 2.14 | 0.029 | 1.11 | 0.76, 1.62 | 0.580 | 1.20 | 0.99, 1.45 | 0.066 |  |  |  |
| **Digoxin** | 1.21 | 0.68, 2.17 | 0.52 |  |  |  | 1.27 | 0.95, 1.68 | 0.1 |  |  |  |
| **PPI** | 1.08 | 0.77, 1.53 | 0.65 |  |  |  | 1.28 | 1.05, 1.55 | 0.015 | 1.09 | 0.88, 1.36 | 0.430 |
| **Antipsychotic drugs** | 1.15 | 0.59, 2.22 | 0.68 |  |  |  | 1.33 | 0.99, 1.80 | 0.059 |  |  |  |
| **Anxiolytic drugs** | 1.47 | 0.84, 2.57 | 0.18 |  |  |  | 1.14 | 0.85, 1.53 | 0.38 |  |  |  |
| **Antiepileptic drugs** | 1.11 | 0.27, 4.53 | 0.88 |  |  |  | 0.93 | 0.47, 1.83 | 0.84 |  |  |  |

*AAD: antiarrhythmics drugs; CAD: coronary artery disease; CI: Confidence Interval; CKD: chronic kidney disease; COPD/OSAS: chronic obstructive pulmonary disease/obstructive sleep apnoea syndrome; DOAC: direct oral anticoagulants; PAD: peripheral artery disease; PAF: Paroxysmal Atrial Fibrillation; PPI: proton pump inhibitor; RAAS: renin-angiotensin-aldosterone; sHR: subdistribution Hazard Ratio; VKA: vitamin K antagonist.*

*†No Cardiovascular Events occurred in CAD group *defined as body mass index≥ 30 Kg/m^2^ **defined as estimated glomerular filtration rate (eGFR)<60 ml/min.*
